# Formation and Stabilization of Gold Nanoparticles in Bovine Serum Albumin Solution

**DOI:** 10.3390/molecules24183395

**Published:** 2019-09-18

**Authors:** Iulia Matei, Cristina Maria Buta, Ioana Maria Turcu, Daniela Culita, Cornel Munteanu, Gabriela Ionita

**Affiliations:** “Ilie Murgulescu” Institute of Physical Chemistry of the Romanian Academy, Splaiul Independentei 202, 060021 Bucharest, Romania; iulia.matei@yahoo.com (I.M.); butamariacristina@gmail.com (C.M.B.); oana_turcu@yahoo.com (I.M.T.); danaculita@yahoo.co.uk (D.C.); cornel_munteanuro@yahoo.com (C.M.)

**Keywords:** gold nanoparticles, albumin, EPR spectroscopy, Raman spectroscopy, circular dichroism

## Abstract

The formation and growth of gold nanoparticles (AuNPs) were investigated in pH 7 buffer solution of bovine serum albumin (BSA) at room temperature. The processes were monitored by UV-Vis, circular dichroism, Raman and electron paramagnetic resonance (EPR) spectroscopies. TEM microscopy and dynamic light scattering (DLS) measurements were used to evidence changes in particle size during nanoparticle formation and growth. The formation of AuNPs at pH 7 in the absence of BSA was not observed, which proves that the albumin is involved in the first step of Au(III) reduction. Changes in the EPR spectral features of two spin probes, CAT16 and DIS3, with affinity for BSA and AuNPs, respectively, allowed us to monitor the particle growth and to demonstrate the protective role of BSA for AuNPs. The size of AuNPs formed in BSA solution increases slowly with time, resulting in nanoparticles of different morphologies, as revealed by TEM. Raman spectra of BSA indicate the interaction of albumin with AuNPs through sulfur-containing amino acid residues. This study shows that albumins act as both reducing agents and protective corona of AuNPs.

## 1. Introduction

The research focused on the synthesis and properties of gold nanoparticles (AuNPs) has expanded into the advanced fields of nanomedicine and nanotechnologies [1,2,3,4]. The interactions of AuNPs with biomolecules found in biofluids are relevant for medicinal applications like imaging, sensing, drug and gene delivery, and in photothermal therapy. AuNPs are usually stabilized in different solutions by layers of small organic molecules. Sulfur, amino or hydroxyl groups bind to the surface of AuNPs through chemical or physical interactions [5]. Citrate, a nontoxic and relevant species for biological systems, represents both a reducing agent and a stabilization agent for AuNPs in water [6]. Albumin can bind spontaneously to citrate-protected AuNPs [7,8,9,10] and the resulting nanosystems are of interest for multicomponent drug system applications.

In recent years, a significant number of studies have reported on the synthesis and properties of AuNPs in the presence of proteins [4,11,12,13,14]. Assemblies of AuNPs protected by lysozyme were tested for loading capacity of hydrophilic and hydrophobic molecules like doxorubicin and pyrene, respectively. These nanocarriers have been then coated with albumin in order to facilitate their uptake by cancer cells [11]. Bakshi et al. [12] studied the biochemical properties of BSA-conjugated nanoparticles synthetized in the presence of ionic surfactants using thermally denatured BSA as reducing agent of Au(III). They found that BSA-protected AuNPs do not show any hemolytic response.

In another study, Murawala et al. [4] reported on the ability of BSA to reduce Au^3+^ at low pH, underlying that reduction did not occur in BSA-free Au^3+^ solution, irrespective of the pH. Interestingly, when both Ag^+^ and Au^3+^ ions are added, Ag^0^ acts as reducing agent for Au^3+^ ions (galvanic exchange reaction) and AuNPs are formed. The authors also infer that Trp residues of BSA and not Tyr residues are responsible for Ag^+^ reduction. The secondary structure of BSA is not altered to a significant extent following its reductive action on Ag^+^ and AgNPs coating. Similarly, in another study, Xie et al. [13] have shown that the reduction of HAuCl_4_ with BSA at physiological temperature leads to the formation of triangular and hexagonal gold nanoplates within two days. The reaction rate and particle morphology depend on the temperature, pH and presence of trace amounts of Ag^+^. The authors concluded that BSA at room temperature has no apparent reduction capability, as no particles were formed within two days.

Basu et al. [14] were the first to report that two proteins, antibodies Rituximab and Cetuximab, can function as reducing and structure-directing agents to produce AuNPs from Au salts. However, they only synthesized AuNPs of 100–1000 nm size and having mostly triangular morphology at acidic pH value. The authors mentioned that experiments at pH 7 and 10 did not lead to the formation of AuNPs. In their analysis, they showed that the type of protecting agent influences the nanoparticle shape. For instance, in the presence of BSA, Au nanotriangles are formed. All NPs were characterized after four weeks of BSA/HAuCl_4_ incubation at room temperature. In the presence of ascorbic acid as an additional, weak, reducing agent, smaller AuNPs (20–100 nm) of flower-like morphology are obtained at pH 3 in a matter of hours.

In this work, we investigated, through a series of physico-chemical methods, the formation of AuNPs using BSA as reducing and protecting agent, at room temperature. The formation and growth of gold nanoclusters have been monitored by UV-Vis spectroscopy, which allowed us to evidence the appearance of the surface plasmon band and the changes in its position. The process also involves protein denaturation, therefore circular dichroism (CD) spectroscopy was used as a tool to prove the changes in albumin conformation. Dynamic light scattering (DLS) and transmission electron microscopy (TEM) gave information on the particle size in solution and in solid state, respectively. Based on the analyses of the Raman spectra of BSA before and after the formation of AuNPs, we evidenced the interaction of sulfur groups from the albumin chain with AuNPs.

**Figure 1:**
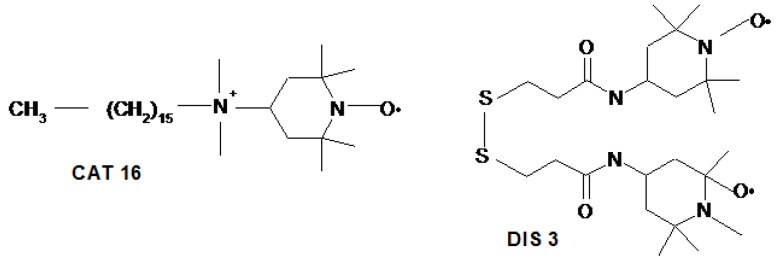
The spin probes used in this study.

Additionally, electron paramagnetic resonance (EPR) spectroscopy was used to investigate the slow process of Au(III) reduction in albumin solution by following the changes in the parameters of the two spin probes presented in Figure 1: 4-*N*,*N*-dimethyl hexadecyl ammonium-2,2,6,6-tetramethylpiperidine-1-oxyl iodide (CAT16) that can report on interactions in various colloidal systems and has affinity for albumin [15,16,17], and a biradical with a disulfide structure (DIS3) that has been used to study the dynamics of ligands at the AuNPs surface [18,19].

Due to its ionic character, CAT16 binds to the albumin binding sites located at the water interface. This spin probe has been previously used to evidence BSA denaturation in the presence of SDS micelles and renaturation following the addition of cyclodextrin [15], to study the interaction of BSA with Pluronic micelles [16] and the thermal denaturation of BSA [17]. We reason that the interaction between albumin and AuNPs can induce changes in the EPR spectrum of CAT16. On the other hand, DIS3 is a biradical with affinity for AuNPs and can thus provide information on the adsorption kinetics on the nanoparticle surface [19].

The investigation of the reduction of Au(III) only in the presence of BSA using a palette of physico-chemical methods evidenced different aspects related to the mechanism of gold nanoparticles formation and their reactivity properties.

## 2. Results and Discussion

### 2.1. UV-Vis Measurements

**Figure 2:**
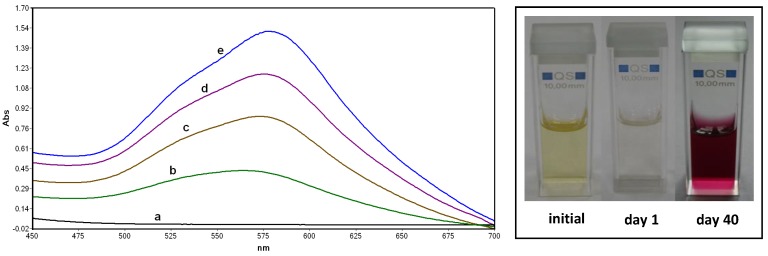
Evolution of the UV-Vis absorption spectrum of the bovine serum albumin (BSA)/HAuCl_4_ system at different stages of incubation: (a) initial, (b) 3 days, (c) 1 week, (d) 2 weeks and (e) 3 weeks. Inset: Color change accompanying the formation of gold nanoparticles.

Reduction of Au(III) was not observed in phosphate solution of HAuCl_4_ (10^−3^ M), but occurred after addition of BSA in a final concentration of 2 mg/mL. The solution underwent a color change from yellow to colorless, corresponding to the reduction process, and then to purple (Figure 2). The reduction of Au(III) in the system containing BSA is due to the presence of specific amino acids in the protein chain, namely 35 threonine and 32 serine units that bear hydroxyl groups [20,21]. The change from colorless to purple color indicates the formation of gold nanoclusters in which the collective oscillation of the metallic surface electrons gives rise to the plasmon resonance band.

The evolution of the reduction process was followed by UV-Vis spectroscopy. It can be observed from Figure 2 that the intensity of the plasmon resonance band of AuNPs increases in time. The optical properties of AuNPs are dependent on the nanoparticle size [22]. The UV-Vis band shown in Figure 2 can be deconvoluted in two components, and this can be an indication that AuNPs have non-uniform size and shape distribution. Assuming a spherical shape of these particles, a particle diameter larger than 20 nm can be estimated from the UV-Vis spectra [22].

### 2.2. TEM and DLS Measurements

The UV-Vis data were correlated with those obtained by dynamic light scattering (DLS) and transmission electron microscopy (TEM). During sample preparation for TEM, the protein was washed out, therefore the TEM images provide information only on the nanoparticle metallic core size.

**Figure 3:**
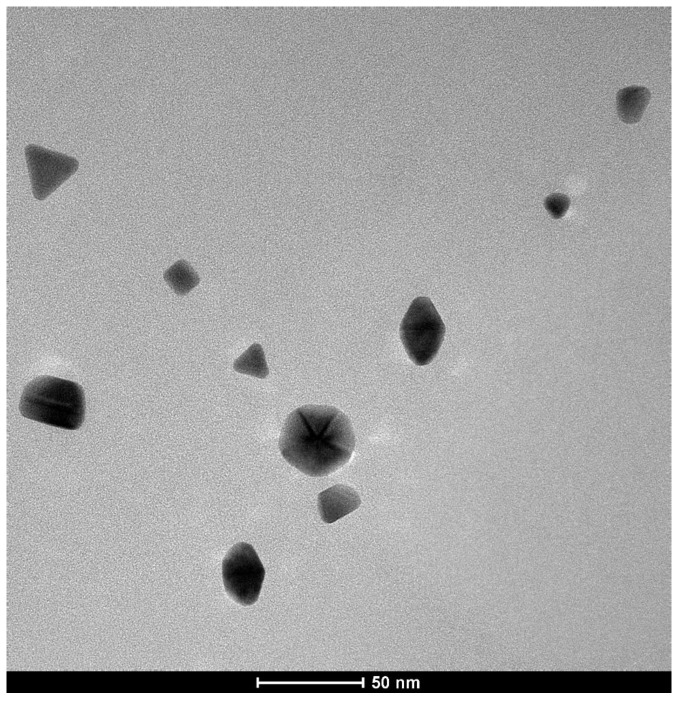
TEM image of gold nanoparticles formed in BSA.

Figure 3 shows the TEM images of AuNPs formed in BSA solution after seven days, evidencing a non-uniform size distribution and the presence of nanoparticles with various morphologies (triangular, rhombohedral, hexagonal; see also Figure S1 in the Supplementary Information file). Other studies [12,20] have also reported triangular and hexagonal morphologies for AuNPs obtained in the presence of BSA. Although the reduction process in the presence of BSA alone was slower compared with the similar processes reported by Bakshi [12] and Xia [20] that use additional physico-chemical factors like temperature, ionic strength and the presence of surfactants, in our case the average particle size of AuNPs was of 20 nm. The dimensions of AuNPs obtained in this slower process were smaller than those reported in the above-mentioned studies. The TEM images were collected at different time intervals and it was observed that, despite a small increase in particle size, the particles remained well-separated, indicative of the fact that aggregation does not occur (Figure S2). Another experiment in which an additional quantity of Au(III) was added to the colorless solution led to the formation of AuNPs with smaller size (less than 5 nm), as shown in Figure S3.

DLS measurements evidence the changes in the dispersion size during the formation of AuNPs. The measurements were performed for a solution of BSA (2 mg/mL) prior to the addition of HAuCl_4_, immediately after addition, and the evolution of the particle size was then followed over the course of one month. Literature DLS data report a size of BSA in solution less than 10 nm [23], a value lower than in our measurements (Table 1). This suggests the presence of BSA dimers in solution. It is important to notice that, in the presence of Au(III), prior to the formation of AuNPs, the size of BSA increases, which might indicate either a denaturation of BSA leading to a less compact protein conformation or the formation of small nanoparticles protected by BSA. DLS measurements performed after three weeks indicated an average particle diameter of 38.6 nm, while after 30 days this value increased to 56.9 nm (Table 1).

DLS measurements indicate also an increase of the nanoparticle size over time. It is possible that BSA plays a role in the growth process of AuNPs, which might be accompanied by conformational changes of BSA that can be revealed by spectroscopic methods.

### 2.3. Circular Dichroism and Raman Spectroscopy

We have shown that BSA acts as reducing agent, but it is also known that albumins can form protective layers for nanoparticles. To demonstrate that BSA is protecting AuNPs and to evidence the conformational changes associated with this process, we performed CD, Raman and EPR experiments.

As it was shown above, the reduction process induced by albumins is quite slow at room temperature. The CD spectra of BSA have been monitored for the entire period of time corresponding to the formation of AuNPs. The extent of BSA secondary structure alteration due to nanoparticle formation can be evidenced from the spectra.

**Figure 4:**
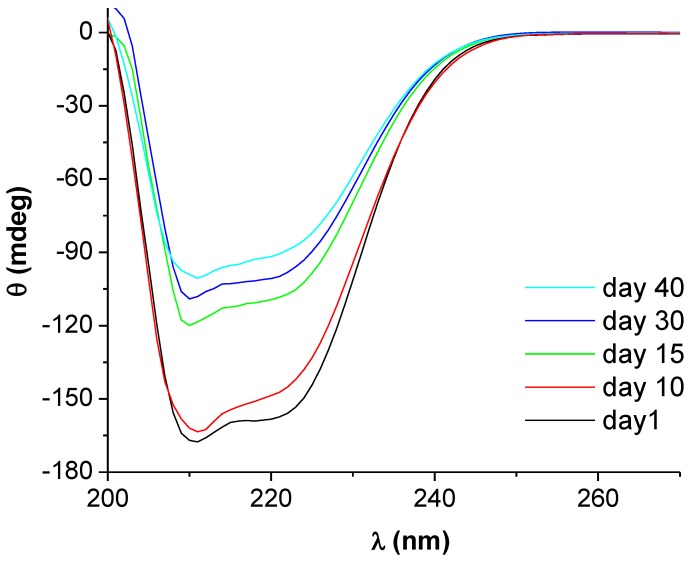
Circular dichroism spectra of BSA (2 mg/mL) recorded at various time intervals and evidencing the alteration of the BSA secondary structure upon gold nanoparticle formation and growth; [HAuCl_4_] = 2 × 10^−3^ M.

The CD spectrum of BSA is characterized by the presence of two negative bands at 209 and 222 nm. These bands arise from π–π* and n–π* transitions in the amide groups, and are typical for proteins with predominantly helical content [24]. Prior to AuNPs formation, the intensity of this signal is only affected by time to a small extent. However, a significant decrease in the CD intensity of BSA occurs upon the growth of AuNPs up to a certain size, as can be seen from Figure 4. We consider that this is the effect of protein corona formation at the AuNPs surface.

The CD spectrum of BSA reflects changes in secondary structure occurring during the reduction of Au(III) as well as during nanoparticle growth. These changes indicate that the reduction step does induce conformational changes in the BSA structure to a lower extent as compared to the growth step, which most probably involves the formation of the BSA corona around nanoparticles with stabilizing effect. Both the reducing and protecting role of BSA induce conformational changes of the protein. As can be observed from Table 2, this consists in the increase of the β-sheet and unordered conformations contents on the expense of the α-helix content.

Further information related to albumin denaturation and AuNPs/albumin interactions is provided by Raman spectroscopy, as will be discussed in the next section.

#### 2.3.1. Modes of Adsorption of BSA onto Gold Nanoparticles

As stated above, the secondary structure of BSA is predominantly α-helical, with the helices being held together by disulfide bridges [25,26]. Protein unfolding, as evidenced by circular dichroism, is also associated to the breaking/reduction in the number of these S-S bonds. The disulfide bridges between Cys residues are a key structural parameter of BSA, as they stabilize the protein’s folded state. Two spectral regions of the Raman spectra of BSA can be used to follow the change in protein conformation occurring upon AuNPs formation: the S–S (480–550 cm^−1^) and C–S (630–720 cm^−1^) stretching modes, sensitive to conformational changes and/or cleavage of the C_α_–C_β_–S–S’–C’_β_–C’_α_ disulfide bridges in BSA [27,28], and the amide I and amide III Raman bands of BSA, indicative of the changes occurring in its secondary structure.

#### 2.3.2. The Configuration of Disulfide Bridges

**Figure 5:**
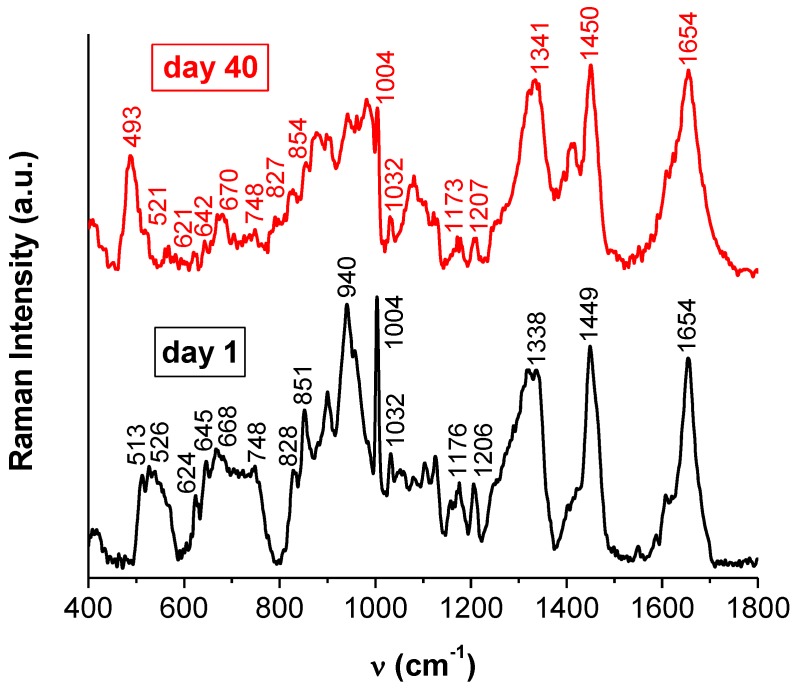
Raman spectra of BSA (20 mg/mL) prior to (day 1) and after (day 40) gold nanoparticle formation; peak positions are marked; [HAuCl_4_] = 4 × 10^−2^ M.

The Raman spectra of BSA prior to and after AuNPs formation are presented in Figure 5. In order to obtain Raman spectra with good signal-to-noise ratio, the concentrations of BSA and HAuCl_4_ were increased compared with those used for other determinations.

The S–S Raman band of BSA includes three main contributions, indicating different configurations of the disulfide bridges: 500–510 cm^−1^ (gauche–gauche–gauche, *ggg*, rotamers), 515–525 cm^−1^ (gauche–gauche–trans, *ggt* or *tgg*) and 530–550 cm^−1^ (trans–gauche–trans, *tgt*) [29,30]. The position of these bands is altered when internal rotation about the S–S and C–S bonds occurs [31].

**Figure 6:**
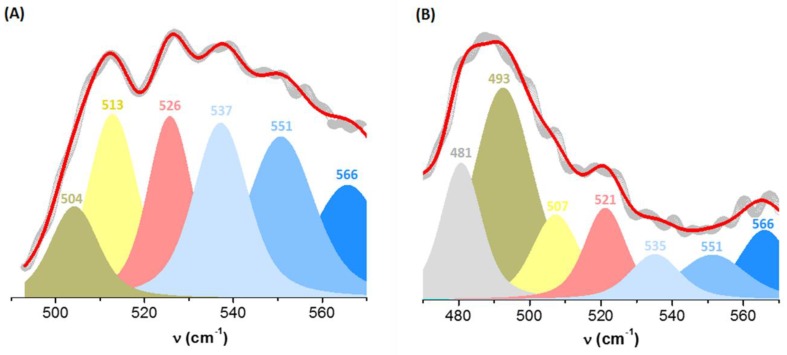
Deconvolution of the Raman bands in the spectral range corresponding to the ν(S–S) vibrations: (**A**) free BSA (day 1, R^2^ = 0.995), (**B**) BSA adsorbed onto gold nanoparticles (day 40, R^2^ = 0.994); the experimental spectra are depicted by grey dots, the convoluted spectra by red lines and the individual component functions are in color.

Band deconvolution in the S–S spectral range yielded six components for BSA (Figure 6A), with an additional component in the presence of AuNPs (Figure 6B). The S–S populations content was computed from the area of the individual components expressed as a fraction of the total area of the bands (Table 3). Initially, the disulfide bridges of BSA assume *ggg* (9%), *ggt* (34%) and *tgt* (57%) configurations. The contributions of *ggt* and *tgt* conformers to the Raman band corresponding to the S–S bond decrease as a result of AuNPs formation, while the bands corresponding to the *ggg* conformer become prevalent and shift towards lower wavenumbers. This indicates that BSA adsorption onto AuNPs favors the more energetically stable S–S conformation, *ggg* [32]. The localization of this band below 500 cm^−1^ indicates that some of these conformers are now found in a restricted medium, the S–S bonds being less flexible [33]. Such bands have been previously reported at 489 [34] and 463 cm^−1^ [35]. Thus, the *ggt* conformation of the disulfide bridges is converted to a *ggg* conformation as a result of protein corona forming onto AuNPs. A similar phenomenon was observed by Nakamura et al. [30] upon BSA conversion from N to F and E forms. The significant enhancement of this vibration in presence of nanoparticles may indicate that the respective disulfide bridges are located near the gold surface, yielding a surface enhanced Raman signal [36]. Differences in peak widths indicate different heterogeneities in the environment of the respective populations [37]. After adsorption, the disulfide bridges are 52% *ggg*, 22% *ggt* and 26% *tgt* configurations.

The reduction in the number of the different S–S bridge populations after AuNPs formation also correlates to changes observed in the features in the ν(C–S) spectral region (Figure 5).

#### 2.3.3. Estimating the Change in BSA Secondary Structure by Raman Spectroscopy

**Figure 7:**
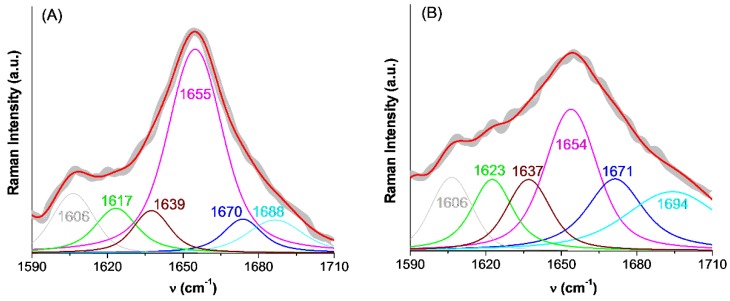
Deconvolution of the amide I region of the Raman spectra of free BSA (**A**, day 1) and BSA adsorbed onto gold nanoparticles (**B**, day 40). The secondary structure contents estimated from the respective peak areas are listed in Table 4.

The analysis of the amide I band in the Raman spectrum provides information on the secondary structure of BSA that can be corroborated with those obtained from CD spectra. Figure 7 presents the deconvolution of the amide I band of BSA initially and after the AuNPs were formed, and the contributions of integrated peaks are listed in Table 4. The integrated peaks correspond to the α-helix (1650–1657 cm^−1^), β-sheet (1612–1640 cm^−1^ and 1670–1690, antiparallel, and 1626–1640, parallel), β-turn (1655–1675 cm^−1^, 1680–1696 cm^−1^) and random coil (1640–1651 cm^−1^) conformations [38]. One observes that both CD and Raman spectroscopies predict the same effect of AuNPs on the helical content of BSA. Moreover, the band at 940 cm^−1^, the skeletal ν(C–C) vibration, another indicator of the helical structure, is intense in free BSA and much weaker when BSA is adsorbed onto the nanoparticles (Figure 5), thus evidencing as well the loss of helical content [30,39].

The Raman data allowed us to reveal some of the specific molecular groups involved in adsorption. Such local changes caused by AuNPs formation and growth can also be evidenced by spin probe method of EPR spectroscopy.

### 2.4. Electron Paramagnetic Resonance Measurements

**Figure 8:**
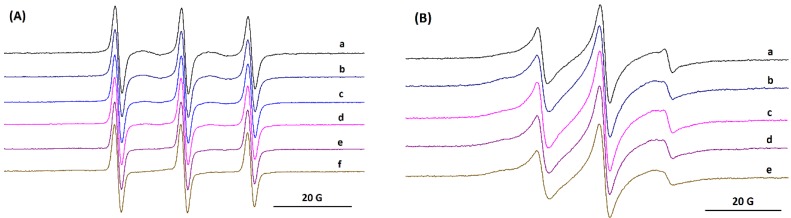
The evolution of the electron paramagnetic resonance (EPR) spectra of (**A**) DIS3: a) initial, b) 4 days, c) 6 days, d) 10 days, e) 13 days, f) 17 days, and (**B**) CAT16 in BSA/Au system: a) initial, b) 4 days, c) 10 days, d) 13 days, e) 17 days.

All species involved in the formation of AuNPs are diamagnetic and, in order to monitor the processes of AuNPs formation and growth in the presence of BSA, we selected the spin probe method of EPR spectroscopy. Thiol derivatives have a high affinity for the Au surface and form protective layers that ensure the stability of AuNPs [40]. Therefore, we selected as a first spin probe the biradical DIS3 (Figure 1) with paramagnetic moieties linked through a disulfide bridge, which has been used for studying dynamic aspects of ligands protecting the Au surface, exchange processes and the stability of AuNPs [18,19,30]. Figure 8A shows the changes in the EPR spectrum of DIS3 during the formation of AuNPs.

In water solution, the EPR spectrum of biradical DIS3 (Figure 8A, spectrum a) shows the additional lines corresponding to exchange interactions (second and forth lines in the spectrum), although their intensities are lower than in organic solvents [18]. The evolution of spectral changes for DIS3 indicates that the lines attributed to the exchange interactions gradually decreased during the reduction step and the formation of AuNPs. After 10 days, the EPR spectrum indicates only three lines, without evidencing a restricted motion (Figure 8A, spectrum d). As it was reported in literature [18,19], adsorption of DIS3 at the surface of AuNPs induces the breaking of the disulfide bond. The changes in the EPR spectrum occur on the same time scale as the changes in the CD spectrum.

The spin probe method of EPR spectroscopy can be used to prove small conformational changes in the BSA structure [15,16,17]. The spin probe CAT16 binds to the BSA sites exposed to water, due to the ionic character of the probe. This determines a relatively fast molecular motion of the nitroxide group in CAT16 as compared to that of 5-doxyl stearic acid, which is almost immobilized in the complex with BSA [16,41,42]. Considering this different behavior of spin probes with affinity for BSA binding sites, we selected CAT16 to analyze the evolution of the BSA/Au system. Figure 8B shows the EPR spectra of CAT16 in solution of BSA in the absence and in the presence of Au, at different time intervals. Spectrum a in Figure 8B presents a double component feature, one with restricted motion and another with fast motion. The formation of AuNPs slows down the motion of the initial faster component, which indicates the fact that the nitroxide group senses the increase in size of the BSA/AuNP assembly (Figure 8B, spectrum e). The simulated spectra of CAT16 in BSA and in BSA/AuNP systems are shown in Figure S4. The simulation of EPR spectra suggests a slower motion of both components once AuNPs are formed. The percentages of the two components corresponding to the EPR spectrum of CAT16 in BSA/AuNPs are approximately the same (Table S1).

**Figure 9:**
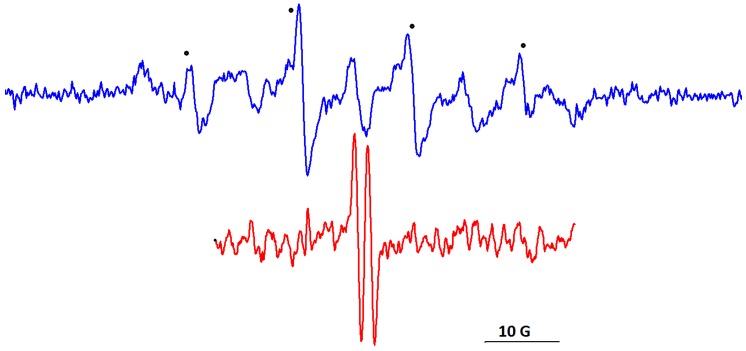
The EPR spectra of 5,5-dimethyl-1-pyrroline N-oxide (DMPO)–HO adduct (in blue) and ascorbyl free radical (in red) generated in the BSA/AuNPs system.

A series of EPR studies reported in literature evidenced that some aerobic oxidations in the presence of AuNPs involve the generation of reactive species [43,44,45]. Starting from this, we tested if reactive radicals that can be trapped by the spin trap DMPO (5,5-dimethyl-1-pyrroline N-oxide) are generated in solution of BSA/AuNPs. In Figure 9, the blue spectrum corresponds to the DMPO adduct with the hydroxyl radical that can be generated in the presence of adsorbed oxygen at the AuNPs surface. The spectrum is a superposition of two components, one corresponding to the DMPO–HO adduct and another corresponding to a nitroxide degradation product of DMPO. The hyperfine coupling constants of the DMPO–HO adduct are a_N_ = 14.85 G and a_H_ = 14.78 G. Generation of hydroxyl radicals in BSA/AuNPs might be significant for the biomedical applications of such systems.

A well-known component of biofluids is ascorbate, a species that acts as radical scavenger. Oxidation of ascorbate is a two-step process that involves the ascorbyl free radical as intermediate. Hydroxyl radicals can generate the ascorbyl radical [46,47,48]. Addition of calcium ascorbate to the BSA/AuNPs solution led to the formation of the ascorbyl radical characterized by a_H_ = 1.78 G, as shown in Figure 9, spectrum in red.

The spin-trapping experiments performed in solution of BSA/AuNPs after purging argon did not lead to the formation of DMPO–HO adducts. The spin trapping measurements suggest that AuNPs might be involved in oxidative processes during their transport to the specific targets.

## 3. Materials and Methods

BSA fatty acid free fraction V was purchased from Fluka (Buchs, Switzerland). Chloroauric acid (HAuCl_4_, >97%) and DMPO were purchased from Sigma Aldrich (St. Louis, MI, USA). These were used without further purification. The solutions were prepared in phosphate buffer at pH 7. The spin probe CAT16 was obtained from Molecular Probes (Leiden, The Netherlands), whereas the spin probe DIS3 was obtained as described in literature [18,19].

### 3.1. Sample Preparation

BSA was dissolved in a solution of HAuCl_4_ (2 × 10^−3^ M) in phosphate buffer at pH 7. The resulting system was left at room temperature over a period of over one month. The concentration of albumin for measurements using different methods were as follows: 2 mg/mL for UV-Vis, DLS, circular dichroism, 10 mg/mL for EPR measurements and 20 mg/mL for Raman spectroscopy.

### 3.2. Instrumentation

#### 3.2.1. UV-Vis Absorption

Absorption spectra were recorded on a Jasco V-560 UV-VIS spectrophotometer in the range 300-700 nm.

#### 3.2.2. DLS Measurements

The particle size evolution in BSA and BSA/Au solutions was analyzed by dynamic light scattering (DLS) measurements in water on a Beckman Coulter particle size analyzer (Brea, CA, USA) using the Delsa Nano software (Beckman Coulter, Brea, CA, USA). The measurements were performed at room temperature.

#### 3.2.3. Circular Dichroism Measurements

Circular dichroism spectra of BSA (2 mg/mL) in the absence and in the presence of HAuCl_4_ (2 × 10^−3^ M) were recorded, at various time intervals, on a Jasco J-815 circular dichroism spectropolarimeter, in 0.05 cm path length cuvettes, at 4 s response time, 100 nm/min scan speed, 1 nm bandwidth and 1000 millidegrees (mdeg) sensitivity. The characteristic dichroic signal of BSA observed in the 200–300 nm spectral range was averaged over six scans with subtraction of the buffer blank. The results were expressed in ellipticity (θ, in mdeg). The BSA secondary structure contents prior to and after nanoparticle formation were determined with the DichroWeb online server [49,50]. The α-helix, β-sheet and unordered contributions were estimated employing the K2D deconvolution algorithm [51]. All fits led to normalized root mean square deviations lower than 0.2 [52,53].

#### 3.2.4. Raman Spectroscopy

Raman spectra of BSA (20 mg/mL) in the absence and in the presence of HAuCl_4_ (4 × 10^−2^ M) were collected on a Jasco NRS-3100 Raman spectrometer with laser excitation at 785 nm (laser power 150 mW). The spectra were averaged over 300 scans and the spectrum of the control (HAuCl_4_ in phosphate buffer) was subtracted from each sample spectrum. Baseline correction and smoothing of spectra with a 15 point Savitsky–Golay function were performed with the Spectra Analysis program. The spectra were deconvoluted to the minimum number of components by fitting the Raman bands of BSA with a sum of pseudo-Voigt functions (Gaussian and Lorentzian, in equal contributions). Band position and width were constrained within reasonable limits.

#### 3.2.5. Transmission Electron Microscopy

The TEM analysis was performed on Tecnai G2-F30 S-twin microscope (FEG-STEM) at an accelerating voltage of 300kV. Sample preparation was minimal, consisting in mounting a drop of AuNPs alcoholic suspension on holey carbon film copper grid allowing the solvent to evaporate at room temperature.

#### 3.2.6. EPR Spectroscopy

EPR spectra of DIS3 and CAT16 were recorded on a JEOL FA 100 spectrometer equipped with a cylindrical type resonator TE011, with a frequency modulation of 100 kHz, microwave power of 0.998 mW, sweep time of 480 s, modulation amplitude of 1 G, time constant of 0.3 s, and magnetic field scan range of 100 G. For the spin trapping experiments, the following settings were used: sweep field 150 G, frequency 100 kHz, gain 800, sweep time 240 s, time constant 0.1 s, modulation width 1 G, microwave power 1 mW.

EPR spectral simulations of CAT16 spectra were performed using the program developed by Budil et al. [54] based on non-linear least–squares (NLLS) fits, allowing fitting of spectra encompassing two components with different dynamics.

## 4. Conclusions

In this study we monitored the formation and stabilization of gold nanoparticles in the presence of BSA by a combination of different physico-chemical techniques. The gold (III) reduction and the growth of gold nanoparticles are processes accompanied by conformational changes in the BSA. DLS measurements evidenced the presence in solution of assemblies with molecular size growing from the size of albumin up to the average size of 40 nm. TEM images evidenced different morphologies of the nanoparticles, with an average size of 20 nm after 10 days and a slow increase to approximately 50 nm over a one-month interval. The results of this study highlight that albumin, as a reducing agent of Au^3+^, can be an initiator for the formation of gold nanoparticles. Moreover, by using EPR and Raman spectroscopies it was possible to prove that albumin covers the gold nanoparticles surface. Using EPR spectroscopy, we were able to monitor the formation of nanoparticles, to analyze the changes in the behavior of spin probes at the surface of albumin, and to evidence the formation of reactive species, intermediated by gold nanoparticles. This physico-chemical study thus provides relevant information for the application of such systems in the biomedical field of nanoparticle delivery.

## Figures and Tables

**Table 1:** Evolution of the average particle diameter in a solution of BSA (2 mg/mL) in the absence and presence of HAuCl_4_ (10^−3^ M).

Sample	Day	Diameter (nm) (Intensity Distribution)	Diameter (nm) (Volume Distribution)
BSA	Initial	20.4 ± 11.9	9.6 ± 4.5
BSA/Au^3+^	Initial	31.1 ± 20.4	12.3 ± 6.3
BSA/Au^0^	21	38.6 ± 19.4	21.5 ± 8.9
BSA/Au^0^	30	73.3 ± 30.1	46.5 ± 17.7

**Table 2:** Secondary structure content of BSA prior to and after gold nanoparticle formation and growth, as evidenced by circular dichroism.

Day	α-Helix	β-Sheet	Random Coil
1	0.60	0.07	0.33
10	0.59	0.08	0.33
15	0.40	0.21	0.39
30	0.36	0.18	0.46
40	0.30	0.18	0.52

**Table 3:** The population of each conformer, calculated as the fraction of its band area from the total area of the bands.

Day 1		Day 40	
ν (cm^−1^)	Area (%)	ν (cm^−1^)	Area (%)
–	–	481	16
504	9	493	36
513	18	507	11
526	16	521	11
537	19	535	7
551	22	551	9
566	16	566	10

**Table 4:** Secondary structure content of BSA prior to and after gold nanoparticle formation and growth, as evidenced by Raman spectroscopy.

Day 1		Day 40		Assignment
ν (cm^−1^)	Area (%)	ν (cm^−1^)	Area (%)	
1617	11	1623	13	β-sheet
1639	9	1637	13	random coil
1655	62	1654	31	α-helix
1670	8	1671	19	β-turn
1688	10	1694	24	β-turn

## Supplementary Materials

The following are available online at https://www.mdpi.com/1420-3049/24/18/3395/s1, Figure S1: TEM images of gold nanoparticles formed in BSA evidencing the presence of various shapes. Figure S2: TEM images of gold nanoparticles formed in BSA after three weeks. Figure S3: TEM image of AuNPs with smaller particle size (roughly 5 nm) formed after adding a new quantity of Au(III). Figure S4: Simulated EPR spectra of CAT16 in (A) BSA (10 mg/mL), (B) BSA/AuNPs after 10 days and (C) BSA/AuNPs after 17 days. Table S1: EPR parameters obtained by spectral simulation.
