# Supplementary material for: Formation and Stabilization of Gold Nanoparticles in Bovine Serum Albumin Solution
Source: Molecules. 2019 Sep 18;24(18):3395. doi: 10.3390/molecules24183395 (PMC6766809; doi:10.3390/molecules24183395)
Supplement: Supplementary file 1 [file molecules-24-03395-s001.pdf]

Formation and stabilization of gold nanoparticles in bovine serum albumin solution

Iulia Matei, Maria Cristina Buta, Ioana Maria Turcu, Daniela Culita, Cornel Munteanu and Gabriela Ionita *

„Ilie Murgulescu" Institute of Physical Chemistry of the Romanian Academy, Splaiul Independentei 202, Bucharest 060021, Romania;

* Correspondence: ige@icf.ro (G.I.)

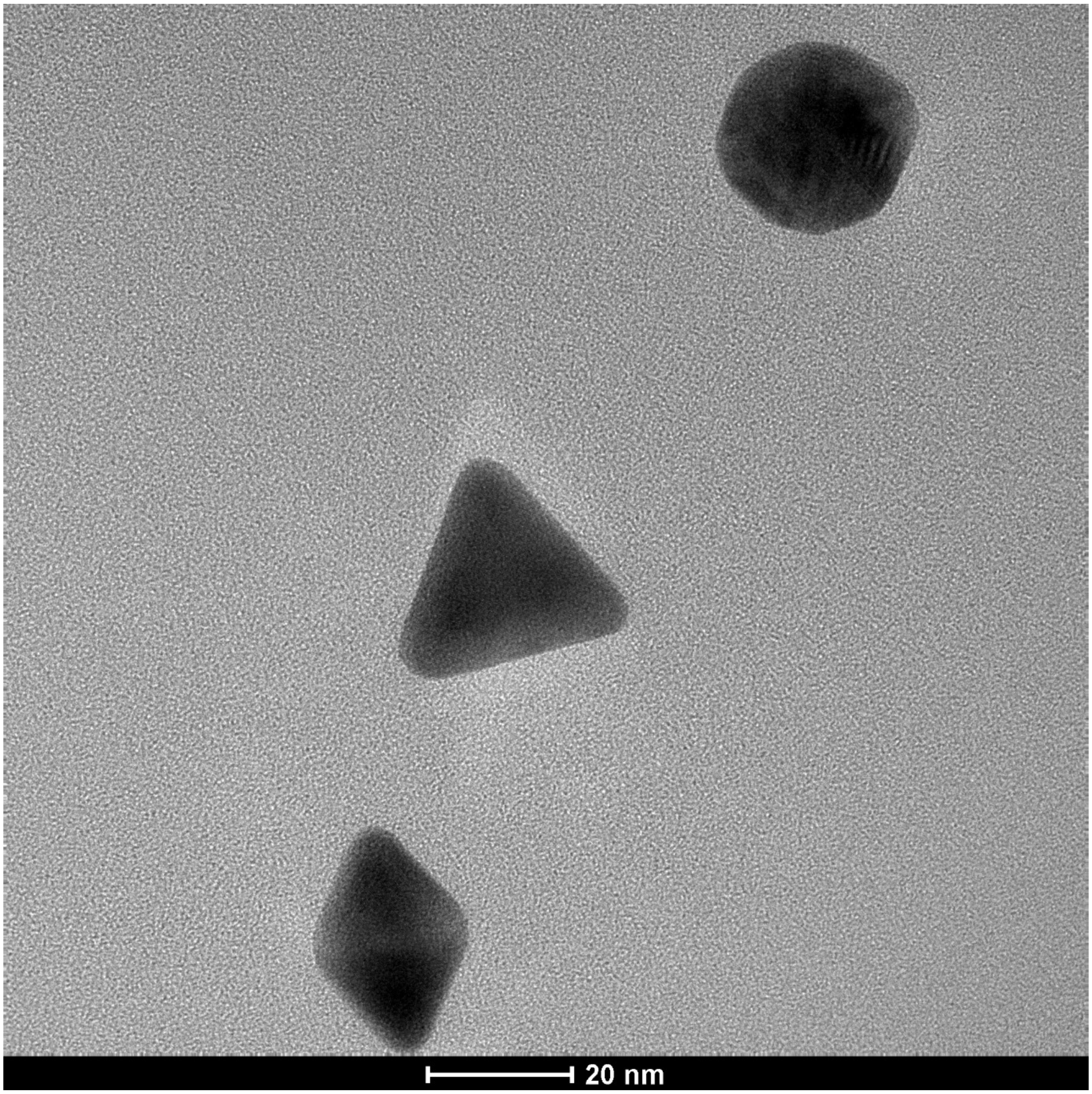

Figure S1. TEM images of gold nanoparticles formed in BSA evidencing the presence of various shapes.

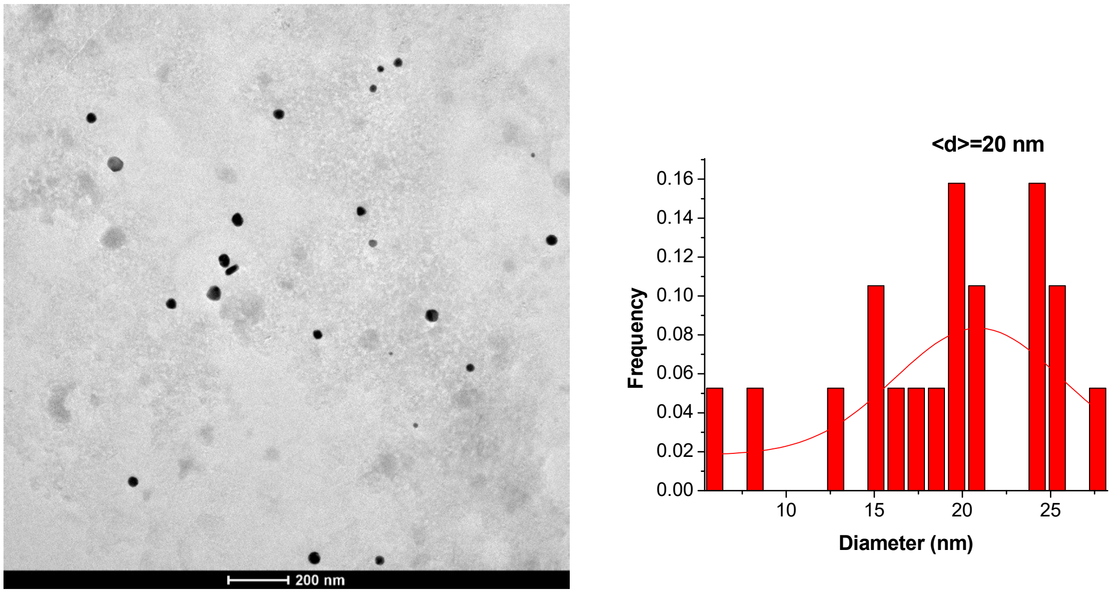

Figure S2. TEM image and size distribution of gold nanoparticles formed in BSA after three weeks.

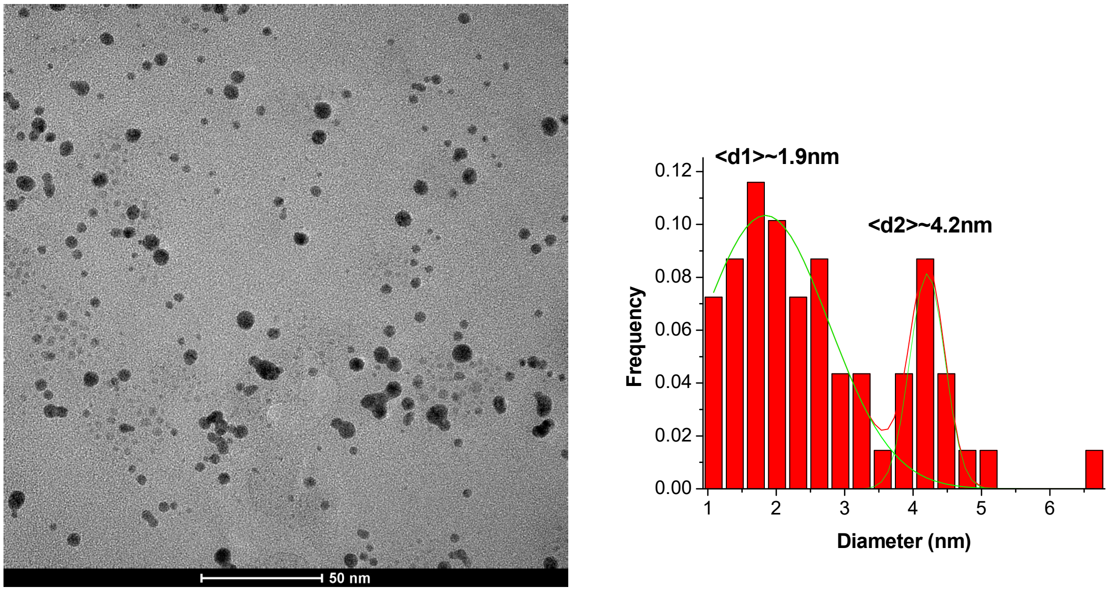

Figure S3. TEM image of gold nanoparticles with smaller particle size (roughly 4 nm) formed after adding a new quantity of Au(III).

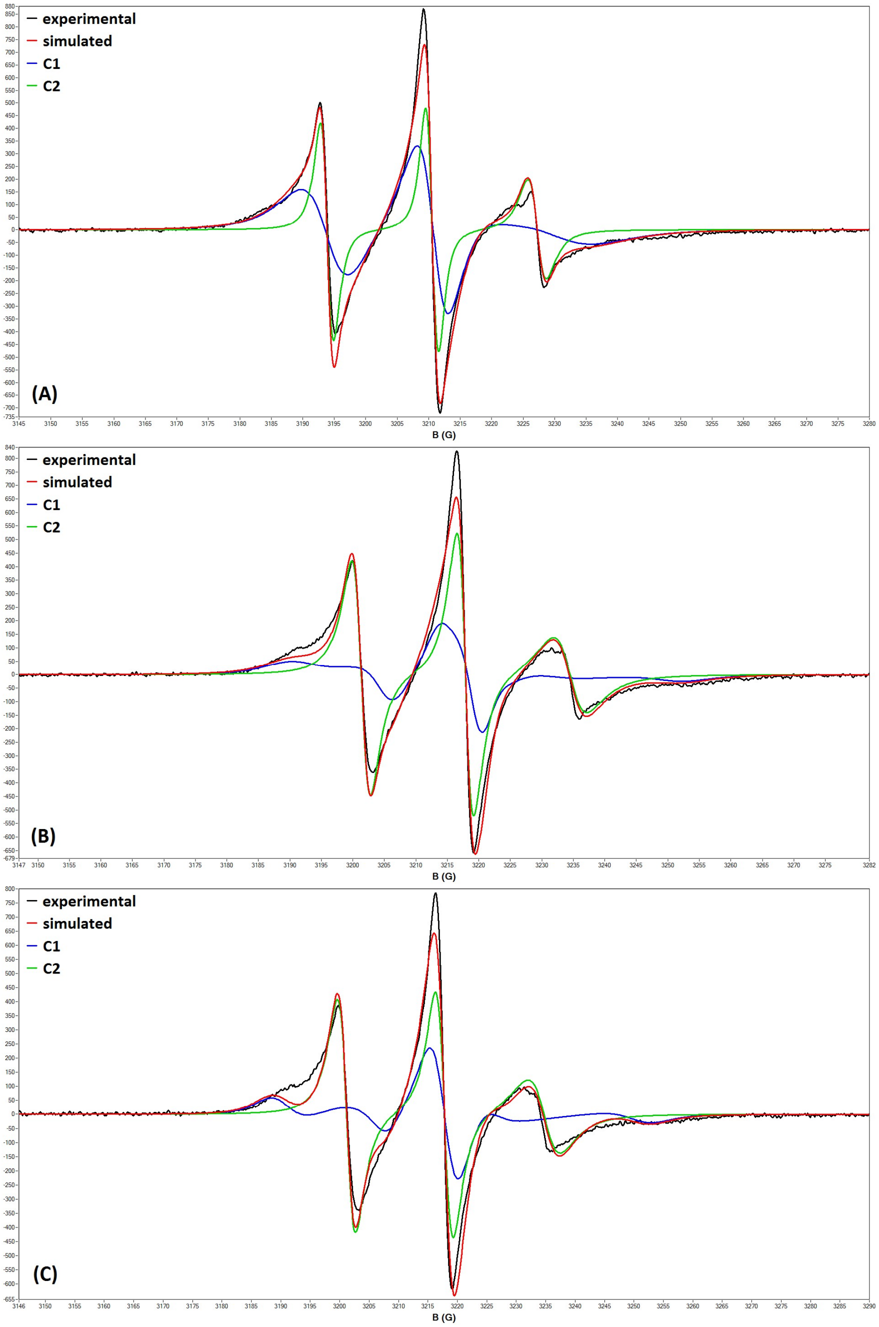

Figure S4. Simulated EPR spectra of CAT16 in (A) BSA (10 mg/ml), (B) BSA/AuNPs after 10 days and (C) BSA/AuNPs after 17 days.

Table S1. EPR parameters obtained by spectral simulation

BSA	BSA/Au^0, 10 days	BSA/Au^0, 17 days
component 1	***component 1***	***component 1***
gxx,gyy,gzz = 2.0085, 2.0056, 2.0035	gxx,gyy,gzz = 2.0085, 2.0056, 2.0035	gxx,gyy,gzz = 2.0085, 2.0056, 2.0035
Axx,Ayy,Azz = 7.0, 7.0, 36.0 (a_N= 16.7 G)	Axx,Ayy,Azz = 7.0, 7.0, 36.5 (a_N= 16.8 G)	Axx,Ayy,Azz = 6.5, 6.5, 36.0 (a_N= 16.3 G)
Rprp = 7.15, Rpll = 8.00	Rprp = 6.40, Rpll = 7.70	Rprp = 7.10, Rpll = 7.20
gib0 = 2.0	gib0 = 2.5	gib0 = 2.5
79.1%	50.8%	48.1%
$\tau = 6.30\times10^{-9}$ s	$\tau = 2.60\times10^{-8}$ s	$\tau = 1.23\times10^{-8}$ s
component 2	***component 2***	***component 2***
gxx,gyy,gzz = 2.0085, 2.0056, 2.0035	gxx,gyy,gzz = 2.0085, 2.0056, 2.0035	gxx,gyy,gzz = 2.0085, 2.0056, 2.0035
Axx,Ayy,Azz = 6.5, 7.0, 36.5 (a_N= 16.7 G)	Axx,Ayy,Azz = 6.0, 6.5, 36.5 (a_N= 16.3 G)	Axx,Ayy,Azz = 6.0, 6.5, 36.0 (a_N= 16.2 G)
Rprp = 8.20, Rpll = 8.75	Rprp = 7.80, Rpll = 8.50	Rprp = 7.70, Rpll = 8.70
gib0 = 1.0	gib0 = 0.5	gib0 = 1.0
20.9%	49.2%	51.9%
$\tau = 6.96\times10^{-10}$ s	$\tau = 1.57\times10^{-9}$ s	$\tau = 1.59\times10^{-9}$ s
R = 0.988	R = 0.986	R = 0.981
